# Dynamic Cancer Cell Heterogeneity: Diagnostic and Therapeutic Implications

**DOI:** 10.3390/cancers14020280

**Published:** 2022-01-07

**Authors:** Valerie Jacquemin, Mathieu Antoine, Geneviève Dom, Vincent Detours, Carine Maenhaut, Jacques E. Dumont

**Affiliations:** Institut de Recherche Interdisciplinaire en Biologie Humaine et Moléculaire (IRIBHM), Université Libre de Bruxelles, 1070 Brussels, Belgium; mathieu.antoine@ulb.be (M.A.); genevieve.dom@ulb.be (G.D.); vincent.detours@ulb.be (V.D.); carine.maenhaut@ulb.be (C.M.)

**Keywords:** heterogeneity, thyroid cancer, molecular level, implication, tumor ecosystem

## Abstract

**Simple Summary:**

Cancer heterogeneity, which occurs in most cancer patients, has been known and studied by experienced pathologists since the early 19th century. Intratumoral heterogeneity between cancer cells can arise from complex genetic, epigenetic, and metabolic modifications under the influence of the tumor microenvironment and confers considerable adaptability to tumors. Despite major advances in cancer therapy, tumoral heterogeneity remains a major obstacle to the successful treatment of cancer. In this review, we highlight the role of intratumoral heterogeneity, focusing on the clinical and biological implications of this phenomenon.

**Abstract:**

Though heterogeneity of cancers is recognized and has been much discussed in recent years, the concept often remains overlooked in different routine examinations. Indeed, in clinical or biological articles, reviews, and textbooks, cancers and cancer cells are generally presented as evolving distinct entities rather than as an independent heterogeneous cooperative cell population with its self-oriented biology. There are, therefore, conceptual gaps which can mislead the interpretations/diagnostic and therapeutic approaches. In this short review, we wish to summarize and discuss various aspects of this dynamic evolving heterogeneity and its biological, pathological, clinical, diagnostic, and therapeutic implications, using thyroid carcinoma as an illustrative example.

## 1. Introduction

Cancer heterogeneity has been known and studied by experimental pathologists since the beginning of the 19th century [1]. First primarily characterized by differences in cellular morphology (1), cancer heterogeneity was refined over the century with the outline of heterogeneity across surface marker expression (2) and later, differences in tumor growth rates (3) and response to therapy (4). Advances in molecular technologies have been instrumental in uncovering the underlying intricate mechanisms of cancer heterogeneity and revealing the true scale of diversity across human cancers.

Tumor heterogeneity refers to the existence of subpopulations of cells, with distinct genotypes and phenotypes which may exhibit divergent biological behaviors, within a primary tumor (intra-tumor) and as well as in its metastases, or between tumors of the same histopathological subtype in different patients (inter-tumor).

**Figure 1:**
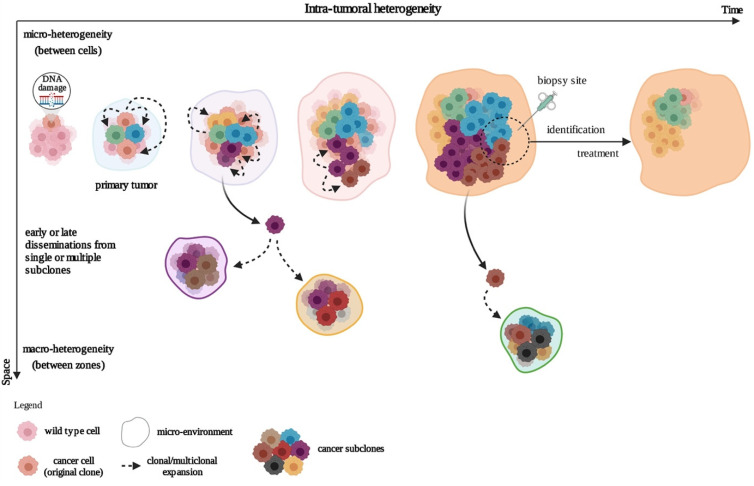
Development of the intra-tumoral heterogeneity. In the clonal evolution model, tumor cells arise from a single mutated cell and acquire additional varied mutations as they progress. This can occur in a linear fashion (not represented) whereby the cells successively acquire mutations that confer a growth or survival advantage, or through a branched mechanism, giving rise to multiple genetically diverse subclonal populations. Created with BioRender.com (accessed on 4 January 2021).

Tumors grow, spread, and co-evolve with their microenvironment, leading to subclonal cell variability in time and space, a notion termed intra-tumoral heterogeneity (ITH) (Figure 1). In time (temporal heterogeneity), variability applies to the dynamic variations in the genetic diversity of an individual tumor which can appear in a relatively short term, depending on changing local conditions (e.g., hypoxia); or in a long term (e.g., genetic, epigenetic). In space (spatial heterogeneity), variability resides in the distribution of genetically diverse tumor subpopulations across different disease sites or within a single disease site or tumor. Evolution of these subpopulations involves underlying genetic, epigenetic, and metabolic features. These subpopulations not only regroup (a) differently evolved cancer cells at different stages, states (e.g., metabolic), and organization levels (e.g., continuous groups, nests, isolated cells, …) but also (b) other cell types such as cancer-associated fibroblasts (CAFs), macrophages, B and T lymphocytes, and endothelial cells, as well as mixtures of these. Strikingly, even neuronal progenitors have been found to infiltrate human prostate primary tumors and are thought to initiate tumor neurogenesis [2].

Spatial heterogeneity also includes the notions of macro- and micro-heterogeneity. Macro-heterogeneity is established by cancer subclones that have undergone significant clonal expansion, while micro-heterogeneity refers to a subclonal genetic diversity observed between neighboring cells, even in one cell type within the tumor. Micro-heterogeneity at the single-cell level is affected by continuous selection, thereby shaping the subclonal cell populations [3]. Spatial and temporal heterogeneity are interlinked to various extents, and may be transient (e.g., metabolic) to permanent (e.g., genetic). These different levels of heterogeneity generate the complex clonal architecture of cancers, fostering drug resistance and inducing a deficient response to therapy [4,5].

The recent pioneering studies on tumor heterogeneity have spurred growing interest, though translation of the concept in both clinical settings [6] (i.e., X-rays, isotopic and ultrasonic examinations, bulk tissue, and circulating DNA measurements) and research (i.e., masked in the study of whole cell populations by molecular biology techniques and in model cancer cell lines) have been slower.

Tumor heterogeneity components should be taken into account, as the genetic pattern found in the primary tumor—which in some cases steers clinical and therapeutic decisions—is based on the major cell population at the point of biopsy. This “partial snapshot” obliviates the fact that it may evolve (a) during tumor progression, (b) in regional or distant metastases, and (c) under evolutive pressure mediated by cancer treatment itself [7]. This leads to the concept of cancer as an independent cooperative heterogeneous cell population with its independently evolving biology; almost an organism [8]. Ultimate examples of this concept are the transmissible cancers in the Tasmanian devil and *canis lupus*, known as devil facial tumor 1 (DFT1) and canine transmissible venereal tumor cancers (CTVT) [9,10], respectively, which have managed to lose immune recognition by mutational events in MHC genes [11].

As personalized medicine becomes more mainstream, we wish to raise the profile of tumor heterogeneity as a key source for challenging effective cancer treatments and of sample bias. This concept raises questions about our present clinical, diagnostic, and therapeutic concepts, as reviewed previously [12] but has not led to a reconsideration of general cancer biology nor of diagnostic or therapeutic concepts.

In this short review, we wish to summarize and discuss various aspects of this dynamic evolving heterogeneity and its biological, pathological, clinical, diagnostic, and therapeutic implications, using thyroid carcinoma as an illustrative example [13].

## 2. Types and Mechanisms of Heterogeneity

ITH between cancer cells may present as genetic or genomic [14], epigenetic/epigenomic [15,16,17], neoantigenic/proteomic [18], and metabolic/metabolomic [19,20], and influenced by the tumor microenvironment (TME) [21,22]. Strikingly, similar heterogeneities also exist in precancerous lesions and in normal tissues but in a milder form [16,23,24], a notion challenging even further cancer therapy.

### 2.1. Genetic and Epigenetic Heterogeneity

Genetic heterogeneity in individual cancer is a result of evolution characterized usually by a monoclonal origin and poly(sub)clonal progression, essentially driven by positive and not negative selection, following local conditions [25,26]. Genetic heterogeneity may also predate the tumor itself, as shown by the widespread prevalence—increasing with age—of oncogenic clones in non-tumoral esophagus [27,28]. The resulting field cancerization [23] and mosaicism therefore represent the extreme non-physiological manifestation of what is the “survival of the fittest” in normal tissue [29,30].

**Figure 2:**
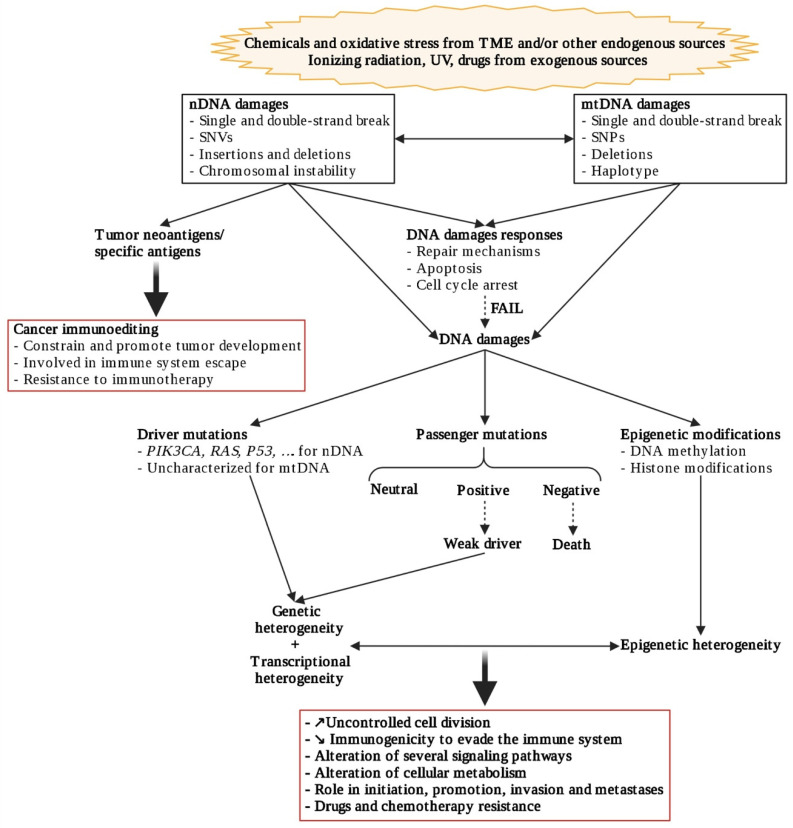
Genetic, transcriptional, and epigenetic heterogeneity communications. Created with BioRender.com (accessed on 2 January 2021).

Genetic heterogeneity involves the accumulation of genetic alterations (Figure 2) such as single-nucleotide variants (SNVs), small insertions or deletions (indels); somatic copy number alterations (CNAs), and structural variants, leading to both branched evolution and elimination of cells and convergent evolutionary process [31]. These genetic alterations predominantly arise from chromosomal instability, itself driven by tetraploidy (e.g., from centrosome aberrations) [32], as from other mutagenic processes. Moreover, mitochondrial dysfunctions may also induce nuclear genome instability and conversely (Figure 2) [33].

Most genetic alterations have a neutral impact (passenger mutations) and may accumulate to 1000 coding substitutions/cell [34], although a weak positive—or even negative—impact on cell survival has been described [35]. In comparison, driver mutations are rare, ranging from around 4 to 10 mutations/cell per cancer, but confer a proliferation/survival advantage. For example, thyroid cancer (TC) cells generally harbor one driver mutation, while colorectal cancer cells can present up to ten [34,36]. However, striking similarities in mutational signatures of different types of cancers illustrate similar evolutionary constraints during their development [37].

As with driver mutations, passenger mutations may appear very early in tumor development [38,39] and their number may increase with treatment, giving rise to multiple subclones [40,41]. It is, therefore, not surprising that during cancer development, genetic heterogeneity may permit immune escape [7] as well as early or late development of polyclonal metastases (Figure 1) [26,42,43].

Moreover, a recent study demonstrated significantly positive correlations between the number of nuclear and mitochondrial DNA (mtDNA) somatic mutations in several cancer types, with the highest correlations observed in chromophobe renal cell carcinoma and TCs. In addition, the authors described the largest proportion of non-silent mtDNA mutations without known nuclear drivers in these cancer types, suggesting a potential functional contribution of mtDNA mutations in the absence of nuclear drivers [44].

As even genetically homogeneous cell populations show remarkable diversity in their response to different environmental stimuli, this suggests that epigenetics can contribute to diversity within tumors (Figure 2) [45]. Indeed, epigenetic gene regulation, which is crucial during normal development to change gene expression patterns, can be also frequently altered by cancer cells to modify their malignant transcriptome and promote tumorigenesis. These modifications involve DNA methylation, post-translational modifications of histones, or changes in higher-order chromatin structures (Figure 2), all with very different time schedules [15,46]. Interestingly, epigenetic ITH calculated from DNA methylation mirrors genetic ITH measures captured at the genomic level [47] in colorectal cancer and glioblastomas, for instance.

Genetic heterogeneity generates a diverse cell population during tumor development and progression, representing a key determinant for variation in tumor therapeutic response and therapeutic failure, with heightened susceptibility of resistance to future therapies.

### 2.2. Transcriptional Heterogeneity

As epigenetic patterns are tightly associated with the transcriptional activity of a cell (Figure 2), human cancers frequently have tumor cell populations with different transcriptional programs, inducing functional heterogeneity [15]. Heterogeneity in different cell populations of tumors is overlooked in global studies such as The Genome Cancer Atlas (TGCA) which excludes tumors with less than 60% of tumor cells (cancers selected for study from http://www.cancer.gov (accessed on 18 June 2021)) and conventional ‘bulk’ RNA sequencing methods which process a mixture of all cells, averaging out underlying differences in cell-type-specific transcriptomes.

However, a whole new chapter of tumor molecular biology is developing, with the rapidly expanding repertoire of single-cell methods enabling cells’ present states to be defined with increasing precision. As demonstrated by single-cell RNA sequencing (scRNA-seq) for solid and circulating tumor cells, heterogeneity at the single-cell level may also result from transcription [8,48,49]. Significant heterogeneity from cell to cell, e.g., of natural killer cells’ cytotoxicity, is also found in apparently homogeneous pathologies such as lymphomas [50].

### 2.3. Tumor Ecosystem: Microenvironment and Metabolism

The whole tumor biology reflects interactions inside cells and from cell to cell. The cells within the TME of solid tumors interact and communicate with each other and with cancer cells, to influence tumor development and progression through metabolites (e.g., collaborations or competition), cytokines, chemokines, growth factors, and inflammatory and remodeling enzymes, for example [21,51,52]. All these interactions evolve in parallel with the tumor cells, inducing a dynamic turnover of the structural and functional components of TME.

#### 2.3.1. CAFs Support Cancer Cell Development

**Figure 3:**
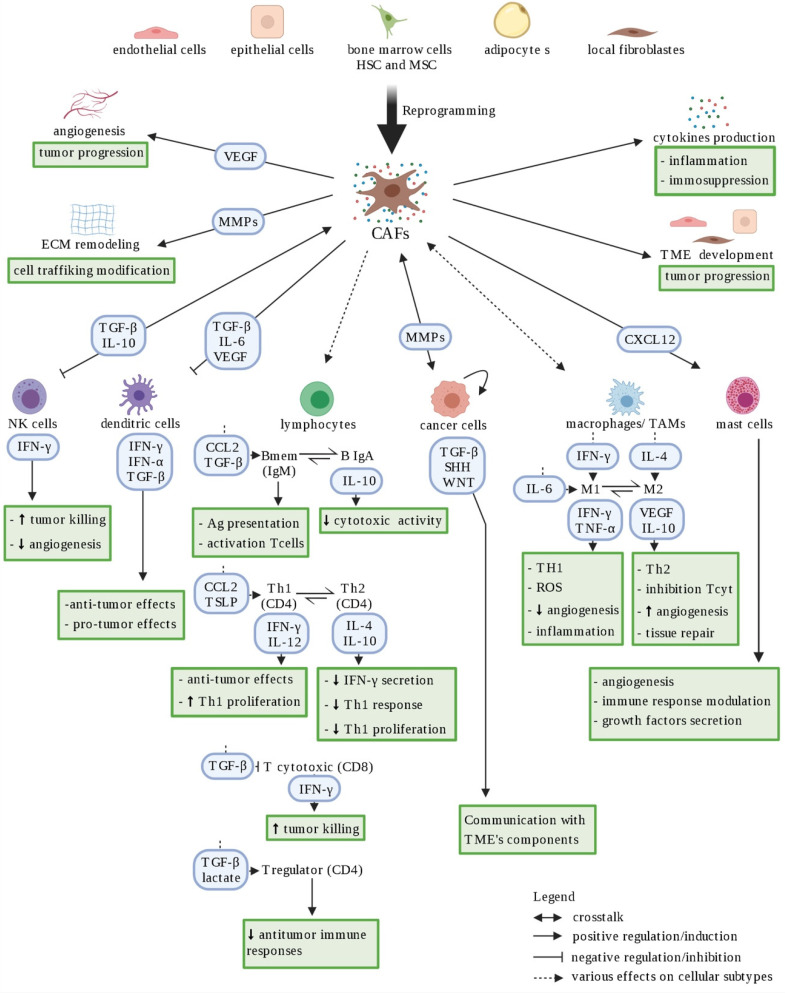
Schematic representation of CAF heterogeneity. Cancer-associated fibroblasts (CAFs) represent a heterogeneous cellular population within the tumor microenvironment, originating from various sources (i.e., epithelial cells, endothelial cells, quiescent fibroblasts, …). CAFs shape the immune microenvironment in tumors toward a pro-tumorigenic and immunosuppressive milieu by affecting the recruitment and function of various innate and adaptive immune cells. Red arrows represent negative regulation/inhibition and blue arrows represent positive regulation/induction. Doted arrows represent a neutral effect. Created with BioRender.com (accessed on 4 January 2021).

CAFs are one of the main group of cells with significant heterogeneity and plasticity in the TME. They can be derived from several cell types, such as resident fibroblasts, epithelial tumor cells, endothelial cells, bone marrow-derived mesenchymal cells, and adipocytes [53] (Figure 3). CAFs play a key role in regulating the biological behavior of tumors by supporting tumor cell survival, dissemination, angiogenesis, immune suppression, and therapy resistance [53,54]. For example, in prostate cancer cells, CAFs promote directional cancer cell migration [55]. They can also provide lactate as substrate for the reverse Warburg effect in papillary thyroid [56] and lung [57,58] cancer cells, and also contribute to the supply of fatty acids to cancer cells, as is the case in breast cancer [59]. Interestingly, inactivation of the secreting transforming growth factor beta (TGF-β) CAFs disrupts oncogenic signaling in transgenic pancreatic cancer cells [60], while other studies reveal potential anti-tumorigenic effects of CAFs [61,62]. In fact, in 2018 Costa et al. identified four subsets of CAFs in breast cancer with different properties (e.g., promoting an immunosuppressive microenvironment) and localization inside the tumor [63], illustrating CAF heterogeneity and offering an explanation to their conflicting role.

#### 2.3.2. Cancer Cells: Immune System “Hijackers”

The immune system plays an influential, albeit dual, role during tumorigenesis. It can both constrain and promote tumor development through a process referred to as immunoediting, proceeding through three phases: elimination (immunosurveillance), equilibrium, and escape. Indeed, various immune cells constituting the TME will variously interfere positively and negatively with tumor progression [64]. The anti-tumorigenic role of the immune systems (innate and adaptive), known as immunosurveillance, naturally targets and destroys transformed and cancerous cells. Briefly, innate immune cells such as natural killer (NK) cells and macrophages recognize transformed cells and produce interferon γ (IFN-γ), while dendritic cells (DCs) secrete interferon α (IFN-α) which will lead to tumor cell death (Figure 3). IFN-γ induces reactive oxygen species (ROS) production; promotes the activity of different immune cells to stimulate antigen presentation, thus generating antigen-specific T cells; activates macrophages towards a more pro-inflammatory phenotype (M1); and inhibits Treg cell differentiation and functions, for example (Figure 3). IFN-α induces expression of tumor suppressor proteins such as transcription factor p53 (TP53). These notions have led to studies implementing various cancer immunotherapies, which use diverse approaches to redirect or hyperactivate the immune system towards the recognition, restraining, and killing of cancer cells [65].

One of the great abilities of cancer cells is to avoid immune destruction by recruiting immunosuppressive cells or through other immunosuppressive mechanisms. This ability is acquired during the equilibrium phase, when any cancer subclone that has survived the elimination phase has increased resistance to immune recognition. The adaptive immune cells, such as T cells, maintain cancer cells in a functionally dormant state to prevent cancer cell outgrowth without destroying them [66]. However, this constant pressure from the adaptive immune system, coupled with genetic instability, leads to the emergence of tumor cell subclones with reduced immunogenicity, which can escape immune recognition and destruction. This selection process occurs through several mechanisms, including: (1) evasion of immune recognition by loss of antigen presentation (absence of MHC class I or co-stimulatory molecules); (2) increase of survival rate (higher expression of anti-apoptotic protein Bcl2); and (3) establishment of an immunosuppressive TME [67]. The latter will be established, for example, by inactivation of cytotoxic T lymphocytes and NK cells through secretion of immunosuppressive factors such as TGF-β and IL-10 (Figure 3) and by expression of inhibitory ligands for the immune checkpoint cytotoxic T lymphocyte antigen 4 (CTLA4) and programmed cell death 1 (PD-1) receptors, such as CD80 and the programmed death-ligand 1 (PD-L1), respectively [66]. In thyroid cancer, malignant thyrocytes are able to counteract the infiltrating immune cells by inducing T-lymphocyte anergy, recruiting Treg, stimulating formation of tolerogenic antigen presenting cells, downregulating neoantigen recognition, and expressing immune checkpoint molecules [68].

Inflammation also has a dual role in anti-tumor or pro-tumor activity, depending on various stages of immune surveillance of the tumor [69]. Interestingly, the inhibitory role of inflammation may be reverted after cancer cell death, and accelerate the growth of surviving malignant cells [70]. Tumors use the activated immune cells in TME to secrete pro-inflammatory cytokines and chemokines, which foster proliferation [71]. For example, inhibition of the pro-inflammatory IL-1β by Canakinumab significantly reduces the risk of lung cancer development [72]. In 2019, Greten and Grivennikov [65] reviewed the role of inflammation during tumor initiation, promotion, and progression. Inflammation has been associated with tumoral development in several cancers, such as thyroid and colorectal cancers, raising critical questions about the role of immune cells in cancer pathogenesis [73,74] even if the development of the majority of cancers and individual tumors is not preceded by long-standing chronic inflammation.

Meanwhile, bulk transcriptional studies and other robust approaches to study the complexities of TME with regard to cellular heterogeneity, precise cell type identification and imaging, as well as cell-to-cell differential transcriptomics, demonstrate enhanced expression of distinct inflammatory cytokines and chemokines in primary tumors and metastatic lesions. These studies also reveal qualitative and quantitative differences in inflammatory cell recruitment [64,75].

#### 2.3.3. Cancer Cells: “Metabolic Parasites”

**Figure 4:**
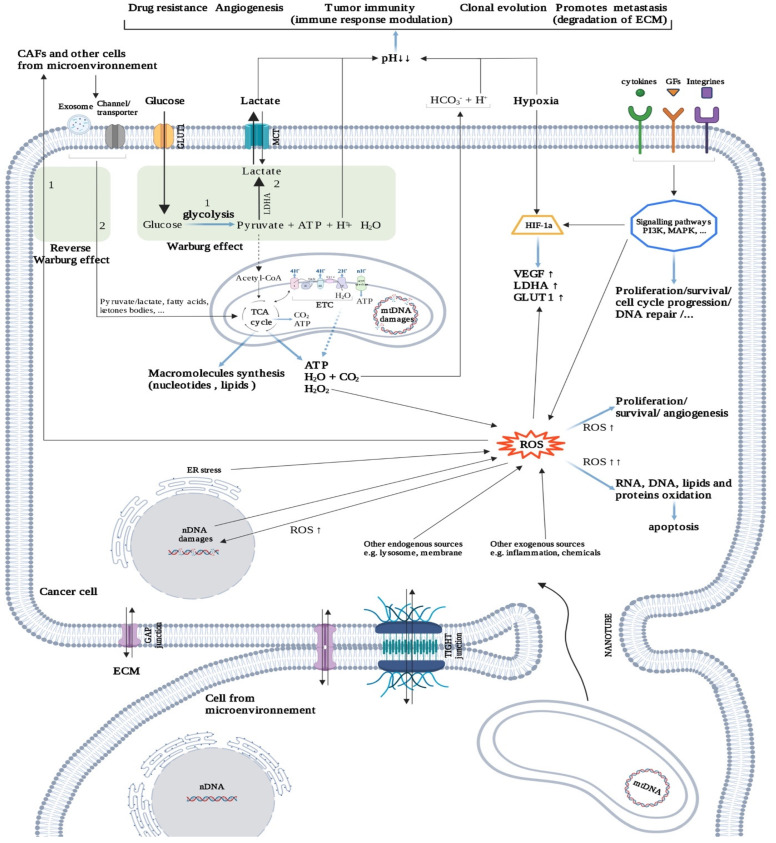
Cancer metabolism. Intercellular communication is facilitated by gap and tight junctions, but also by nanotubes, exosomes, and interleukins. Cancer cell metabolism mainly uses glucose through the Warburg effect, which increases acidosis in the tumor microenvironment (TME). Acidosis triggers several mechanisms for tumor survival. Under hypoxia conditions, the cancer cell can restart the oxidation chain (TCA cycle; Krebs cycle, ETC; electron transport chain) in several ways (e.g., reverse Warburg effect, mitochondrial transfer). Hypoxia promotes the formation of ROS, which play, depending on their concentration, a variety of roles in tumor progression (positive and negative). Created with BioRender.com (accessed on 2 January 2021).

Most cancer cells exhibit a metabolic reprogramming to enhance glycolysis rate even in presence of oxygen, rather than oxidative phosphorylations, to produce adenosine triphosphate (ATP), termed the “Warburg effect” or aerobic glycolysis (Figure 4). Glycolysis also provides precursors for anabolic purposes (e.g., synthesis of amino acids, nucleotides, and lipids, etc.) but also reduces the ATP production level. Cancer cells are thought to undergo a real “rewiring”, using waste from normal cells to produce biomass [76]. Indeed, cancer cells take up high-energy metabolites such as lactate, ketone bodies, free fatty acids, and glutamine from supporting cells (reverse Warburg effect) (Figure 4), including CAFs, cancer-associated adipocytes (CAAs) [77], and myeloid-derived macrophages [78].

Technological advances in the measurement of metabolic parameters at the cell level made possible the identification of very heterogeneous behaviors depending on the cell location in the same tissue. Indeed, expression of heterogeneity between regions of a tumor is seen in colon cancers where MAPK pathway is activated in peripheral cells while NOTCH pathway is activated in central cells. This phenomenon may be regulated, enhancing one type and leading to inhibition of the other, and conversely, suggesting that this is a mechanism by which these tumors resist treatment [79].

In addition, the dysregulation and readaptation of mitochondrial functions remains one of the main components of metabolic reprogramming (Figure 4). (1) Their ATP production is reduced and becomes mostly dedicated to producing anabolic precursors through the tricarboxylic acid cycle. (2) They provide building blocks for anabolism (such as amino acids) via anaplerosis, and (3) produce ROS which play a role in redox homeostasis and oncogenic pathways. Finally, (4) mitochondria are also able to control intrinsic apoptosis. For example, in human head and neck cancers, cell proliferation is strictly correlated with oxidative mitochondrial metabolism [80]. Furthermore, this mitochondrial-dependent metabolism also modulates tumor macrophage function and conversely [62,78].

Parallel to the intracellular modification of metabolism pathways, there are several communication mechanisms between cells to support tumor growth. For example, some cancerous cells express channels that allow normoxic cells to supply hypoxic neighbors with acid-neutralizing HCO_3_^-^ ions [81].

Intercellular communication largely relies on information exchange via: (1) indirect communication through soluble factors which can promote or not tumor development (e.g., interleukins, exosomes, microRNAs); (2) direct cell–cell contacts (e.g., tunnel nanotubes for mitochondria transfer, cellular junctions for cell homeostasis); and (3) cell–extracellular matrix (ECM) crosstalk (e.g., fibronectin for adhesion modification) (Figure 4). More distantly, lung adenocarcinoma activates, without metastasis, osteoblasts in bone marrow which reciprocally supplies it with neutrophils [82]. This and other peripheral effects may even favor metastases, although it should not necessarily be interpreted in a finalist way as distant “niche” preparation, such as in the metastasis itself [83]. Remarkably, intercellular collaboration in cancer may be extended to bacteria and fungi, which may even accompany the cancer cells in metastases [84].

The role of O_2_ supply and anoxia in cell interrelations is also major. Both chronic and cycling hypoxia induce new cancer features [85,86], including cell heterogeneity [87]. Indeed, the ROS produced from the same oxygen may have opposite roles on cancer cells (Figure 4), both early (promoting proliferation) and late (promoting apoptosis) in tumor evolution [88]. As a tumor develops and its blood perfusion becomes ineffective, diffusion distances increase, and the environment surrounding cells in under-perfused areas becomes hypoxic and acidotic. Under hypoxia, the final balance of glycolysis, including breakdown of generated ATP, is the production of lactate and a stoichiometric quantity of H^+^ ions [89]. In the more oxidative tumor areas, the tumor cells also produce H^+^ ions resulting from the hydration of CO_2_ produced from aerobic respiration [89]. Such metabolic shifts have been reproduced in in vivo whole-tumor experimental models [90]. This ambient acidosis has several consequences on the cancer cells’ physiology, such as (1) adaptation of tumor metabolism by reducing glycolysis while promoting mitochondrial activity fueled by fatty acid oxidation [89], which allows the cells to grow in acidic media; (2) inducing a remodeling of the ECM; and (3) promotion of local invasion and metastasis [91] (Figure 4).

All these mechanisms induce a metabolic heterogeneity which varies across time, allowing cancer cells to adapt according to the availability of different nutrients and to the local microenvironment conditions such as acidosis (which inhibits glycolysis) and hypoxia [92,93]. For example, in human lung tumors, the choice of preferred substrate by an area reflects the perfusion rate [94], while in breast cancer it may result from local paracrine signaling [95]. On the other hand, as tumors grow they converge on similar biological characteristics (i.e., sustained proliferation, invasion, immune modulation) through intercellular communications (i.e., interleukins, exosomes, and mitochondrial transfers), which will tend to unify cancer cell phenotypes [96].

## 3. Thyroid Carcinoma as an Example: Various Patterns of Heterogeneity

Thyroid carcinomas (TCs) represent the most frequent tumors among the endocrine neoplasms. With their broad heterogeneity, TCs represent one of the most remarkable examples of variabilities amongst all malignancies. TCs can be subdivided into three main histological types: differentiated (DTC), including papillary, follicular and medullary thyroid carcinoma (respectively PTC, FTC, and MTC); poorly differentiated TC (PDTC); and undifferentiated anaplastic thyroid carcinoma (ATC). Each TC type has several variants which impact on the subtype, such as, for example, aggressiveness [13]. FTC and PTC make up about 95% of all thyroid cancers, and with ATC (around 1% of TC) derive from malignant transformation of follicular cells [74]. MTC, which represents about 3% of thyroid carcinomas, derives from the parafollicular C cells [74]. Contrary to ATC, papillary and follicular thyroid carcinomas have low overall mortality but are likely to relapse.

Thyroid carcinomas present good models for other tumors, with their easy morphologic distinction, their well-identified respective primary oncogenic pathways, and their cell type heterogeneity. With a low proliferative rate and high genome stability, PTC is largely homogeneous with clusters of subclones (zonal heterogeneity), whereas FTC presents a higher degree of heterogeneity, resulting in a mixture of cells in the same cluster (cell-to-cell heterogeneity) [97], and ATC being the most heterogeneous of all TC subtypes [13]. Moreover, these tumors are easily accessible for biopsies and imaging, and are long-evolving with regards to PTC and FTC.

The cell heterogeneity in TCs mostly involves CAFs, tumor-associated macrophages (TAMs), lymphocytes, and endothelial cells, but their abundance can vary between the three cancer types [13,98].

Several studies have revealed immune cell infiltration and immune checkpoint expression in thyroid cancer [74]. The nature of immune cells infiltrating thyroid tumors has to date been poorly studied, even if many studies have confirmed the high expression of CD4+ and CD8+ T cells [71].

**Figure 5:**
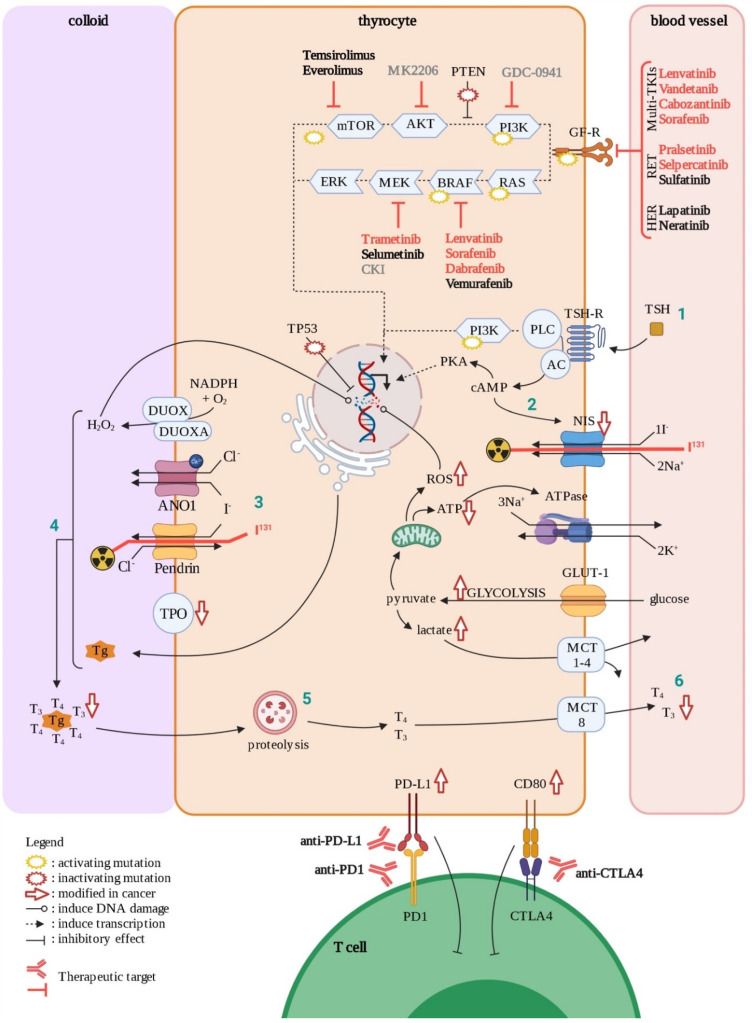
Thyroid cell in normal and cancer situations. In normal condition, thyrocytes produce thyroid hormones (T3 and T4) in response to TSH through several steps (1–6). During oncogenesis, mutations (with positive or negative effects on clone selection) and other factors will affect physiological functions in the cell (symbolized by red arrows). Approved treatments are in red, ongoing clinical trials are in black, and experimental drugs are in grey. GF-R; growth factor receptors, AC; adenylate cyclase, Tg; thyroglobulin, TPO; thyroid peroxidase. Created with BioRender.com (accessed on 4 January 2021).

TCs also represent an example of the role of oxidative stress in tumorigenesis through its impact on genomic instability. Indeed, in normal conditions, follicular thyroid cells synthesize thyroid hormones, triiodothyronine (T3) and thyroxin (T4), through a complex mechanism that requires production of H_2_O_2_ generating an oxidative stress (Figure 5) [99]. Dysregulation of the control of H_2_O_2_ production, as well as other ROS accumulation, is considered to be a cause of DNA damage, which is in turn an initial step of tumorigenesis. However, compared to other tumors, TCs show a low overall density of somatic mutations, suggesting that DNA repair has an important role in the maintenance of DNA integrity. The genetic lesions and mechanisms causing these tumors have been summarized [100,101].

### 3.1. Papillary Thyroid Carcinomas

PTC is the most common type of thyroid cancer, accounting for approximatively 85% of TCs. They are moderately aggressive, proliferating, relatively dedifferentiated tumors caused by activation of the MAPK and PI3K pathways, through several driver mutations. Among these, close to 70% are point mutations of *BRAF* (the most prevalent of which is *BRAF^V600E^*) or *RAS* and *RET–PTC* translocations (Figure 5) [100]. In addition, deregulation of CTLA-4 ligands (CD80 and CD86) and PD-1 ligands (PD-L1/2) gene expression have been observed in both PTC and ATC tissues, permitting tumor immune escape (Figure 5) [102]. Although most PTC driver mutations are clonal, a portion occurs subclonally, making PTC a good example of subclonal evolution [103].

These tumors mainly invade lymphatic vessels and generate regional lymphatic metastases [100,104]. Distant metastases are infrequent, although lung and bone metastases have been described, and their development is associated with a poor diagnosis [105].

In PTC, cancer cells are clearly different from normal cells due to distinctive nuclear features, with no intermediate transition forms between them [106]. PTC presents the highest heterogeneity in the stroma among the different TCs, therefore representing a good example of qualitative cell heterogeneity. Their microenvironment is constituted of up to 50% of fibroblasts in distinct, close areas, which may proliferate at the same rate as the cancer cells [107], and of an important proportion of immune cells [108]. Interactions between cancer and stromal cells can be addressed experimentally. A PTC mouse model in which thyroid-specific expression of oncogenic BRAF is activated and PTEN is lost (BRAF^V600E^/PTEN^−/−^/TPO-Cre), displays a higher density of CAFs in the TME [109].

Metabolically, PTC exhibit a reverse Warburg phenotype with mitochondrial activity, in part supported by lactate provided by the CAFs, and anaplerosis [110] as shown in breast cancer cells [111] and others [112].

### 3.2. Follicular Thyroid Carcinomas

FTC is the second most common type of thyroid carcinoma, accounting for around 10% of TCs. FTCs are moderately aggressive, relatively dedifferentiated tumors caused mainly by activating mutations in gene coding for proteins involved in the PI3K–AKT pathway (Figure 5) [100]. They have many features in common with benign follicular adenomas, with quantitative rather than qualitative gene expression differences, suggesting a biological continuum [113]. This suggests the progressive addition of mild genetic lesions to the initial one in effectors of the PI3K pathway (*RAS* mutations found in 28–68% of FTC) [100]. These tumors tend to spread mainly through blood vessels [104] and metastasize, most commonly to lungs and bones [114].

### 3.3. Anaplastic Thyroid Carcinomas

Although ATCs account for less than 2% of all thyroid cancers, their aggressive clinical behavior and intrinsic resistance to radioactive iodine and other therapeutic approaches are responsible for 40–50% of TC-related deaths, even if radically resected at an early stage. Indeed, in addition to considerable local invasion, ATC spreads rapidly to regional lymph nodes and distant sites (mainly lung and bone). Contrary to some other thyroid carcinomas, ATC does not respond well to conventional therapies [115].

ATC may derive from both papillary and follicular carcinomas or from normal thyroid tissue, by qualitative, as of yet undefined mechanism(s), although a separate fetal origin has been suggested by Takano et al. [13,116]. This fetal cell carcinogenesis hypothesis, in which mutational events may occur in thyroid precursors at different stages of differentiation, has been extended to all TC subtypes [117]. However, it has been widely observed that most of the patients with ATC have previous or concomitant differentiated thyroid carcinomas, and an “anaplastic transformation” from the differentiated carcinoma to the anaplastic carcinoma is sometimes clinically observed. In addition, ATC share mutations with PTC and FTC and show largely overlapping mRNA expression profiles with most genes regulated in ATC being also regulated in PTC, and with many of the modulations observed in PTC amplified in ATC [118]. The sudden onset and explosive course of ATC remain poorly understood; however, single-cell analyses could give some insight on the unknown mechanism(s) of this transition from slow-growing to explosive tumor.

ATC is the most heterogeneous of all TCs subtypes regarding (1) the morphology of the cancerous cells and (2) genetic alterations [13]. Mutations in *TERT* promoter and *TP53*, as well as alterations of the PI3K/AKT pathway, are more frequent in ATC (Figure 5). In addition, BRAF mutations, in particular the *BRAF^V600E^* point activating mutation, occur in approximately 45% of ATCs [119]. Less typical mutations can also affect gene coding for

proteins involved in cell cycle regulation and genes associated with histone modifications [100].

ATCs have the highest density of TAMS in their TME, which correlates with poor survival rates [120], although the importance of CAFs on thyroid cancer progression must not be forgotten [121]. The inclusion of this very important population of immunosuppressive, proangiogenic macrophages by ATC fits in well with the general concept of a locally healing permanent inflammation [78].

### 3.4. Diagnosing Thyroid Cancer

The accurate diagnosis of thyroid cancer is important for effective clinical management. To date, fine-needle aspiration (FNA) and/or surgical biopsy followed by cytological analysis represents the most reliable procedure to diagnose thyroid cancer. Though FNA is highly specific for thyroid cancer, it harbors a lower sensitivity, with up to 30% of nodules detected as indeterminate lesions [122], underscoring the need for the discovery of additional markers.

Molecular markers such as T3, T4 and TSH levels, which give insight on thyroid function, but also tumor markers can be analyzed from blood samples. The latter have become a potent tool in TC management to distinguish benign from malignant lesions; to predict aggressive biology, prognosis, and recurrence; and to identify novel therapeutic targets. Obtained from biopsy samples as well, these tumor markers include (1) different mutated genes such as BRAF, RET/PTC, or PI3K/AKT signaling pathway genes, for example, but also (2) several microRNAs and long non-coding RNAs which define a “specific” pattern between the different thyroid cancer subtypes. Moreover, (3) protein expression profiles have also been used in TC diagnostics, but their clinical applicability has yet to be assessed. All these molecular markers able to guide the TC management were reviewed by Nylén et al. in 2020 [123].

In recent decades, in order to decrease the risk of surgical biopsy and to aid in diagnosis, several imaging techniques have been developed and used to detect cancer masses such as computed tomography (CT-scan), positron emission tomography (PET-scan), magnetic resonance imaging (MRI) and radioiodine scan.

A better understanding of thyroid cancer heterogeneity, enabled by new technological advances will undoubtedly impact thyroid cancer management, as reviewed by Tarabichi et al. [124].

### 3.5. Current and Future Therapies for Thyroid Cancer

Despite the favorable prognosis of this disease, around 20% of DTC cases and most anaplastic types remain resistant to standard treatment options, including thyroidectomy, external radiotherapy, and radioactive iodide (RAI, I^131^ in Figure 5). In addition, around 30% of MTC cases show resistance after surgery. The evolving understanding of disease-specific molecular therapeutic targets has led to the approval of several multi-tyrosine kinase inhibitors (multi-TKIs) for RAI refractory DTC such as Sorafenib (VEGFR/PDGFR and Raf), and Lenvatinib (VEGFR/PDGFR/FGFR/RET), and for MTC such as Vandetanib (EGFR/VEGFR/RET inhibitor) and Cabozantinib (VEGFR/RET) [125,126] (Figure 5). Ongoing clinical trials for multi-TKI inhibitors in different thyroid cancer subtypes were extensively reviewed in 2020 by San Román Gil [127].

The current knowledge of involvement of the PI3K and MAPK pathways in thyroid tumor cell pathogenesis has led to the study of several components of these pathways. For instance, two inhibitors of mTOR (Temsirolimus and Everolimus), used for treatment in advanced renal carcinoma, are currently on clinical trials for thyroid cancer (https://clinicaltrials.gov/ (accessed on 27 September 2021)). Multi-kinase inhibitors targeting the MAPK pathway, such as Vermurafenib (BRAF) and Selumetinib (MEK) are under study for advanced thyroid cancers, though none of them have yet been approved by the FDA. However, in 2018, two other inhibitors of this pathway, Dabrafenib (BRAF) and Trametinib (MEK), were approved in the treatment of BRAF-mutated advanced thyroid cancers.

Other therapeutic approaches targeting immune escape for example, previously approved for treatment of other types of cancers (i.e., melanoma and renal carcinoma), are currently investigated in clinical trials for thyroid cancer antibodies against CTLA-4 such as Ipilimumab (in combination with Nivolumab and Cabozantinib) and Tremelimumab (in combination with Durvalumab), and antibodies to PD-1 such as Pembrolizumab (alone or in combination with Lenvatinib or other drugs) and Nivolumab (https://clinicaltrials.gov/ (accessed on 27 September 2021)) [125].

In thyroid carcinoma, more general treatments are still useful, such as those acting on targets at the common beginning or end of the causal chain: BRAF and MAPK inhibitors for papillary carcinoma or PI3K inhibitors for follicular carcinomas [13]; and in other cancers, drugs acting on the early driving mutations [39]. At the end of the causal chain, inhibitors of cyclin dependent kinases, which act on 50% of the presently incurable ATCs (J. Pita unpublished) and on advanced breast tumors, as well as chemotherapy and radiotherapy, are other unspecific “non-personalized” promising classes of treatments. A number of studies [128,129,130] which assessed HER2 expression in thyroid cancer have shown that a substantial percentage exhibit the increased presence of HER2/3 receptors, with expression levels comprised between 40% (FTC) and 18% (PTC) [131], indicating HER2/3 as a potential therapeutic target. Similarly, a common consequence of cancer—in this case dedifferentiation—can be targeted in TC by redifferentiation and reinduction of iodide transport and radioiodide uptake by TSH [132] or cAMP generation.

## 4. Conceptual, Diagnostic, and Therapeutic Implications of the Dynamic Heterogeneity of Cancer

Tumor evolution depicts changes in tumor heterogeneity along the temporal axis and describes the dynamics by which, under environmental and/or treatment pressure, subclonal populations of cancer cells bearing selective advantages emerge at the expense of others. The timing of heterogeneity will vary much depending on the mechanisms involved. While metabolic parameters or substrate availability may vary in hours or days, epigenetic events, cell recruitment, or metabolic patterns (e.g., Warburg or reverse Warburg, preferred substrate, etc.) will require at least weeks, with genetic events and cell recruitments having consequences in months and years. The concept of heterogeneity of cancer and its evolution have implications on our view of cancer, on its diagnosis, and on its therapeutics.

### 4.1. Conceptual Point of View

The dynamic of cancer heterogeneity leads to differences depending on the approach and the methodology used to study it. Clinicians, radiologists, and users of in vivo visualization methods (except, to some extent, nuclear medicine and MRI practitioners), as well as clinical biologists, geneticists, and molecular biologists, mostly consider the tumor as a whole. Similarly, experimental researchers using in vitro cell lines or transgenic animals study homogeneous models, most often fixed at one point in time. These are studies on an assumed homogeneous disease rather than on a heterogeneous tumor. Indeed, tumors are composed of mosaics of cancer cells with different characteristics attributed to both genetic diversity and non-genetic influences. Pathologists, through their examination of tissue samples, have a much more varied vision of the tumor in space, from zone to zone, and even from cell to cell. As there is little communication between the two categories of disciplines, efforts should be made to form multidisciplinary teams to ensure a more holistic approach in tumor analysis at the genetic, molecular, and clinical-radiological level.

Single-cell DNA and RNA sequencing will further enlarge the molecular complexity at one point in time. This is starting a whole new field of tumor biology at the cell level by integrating, without any prior model, thousands or millions of data, to derive a few defining factors which may allow to propose adequate approaches [8,133]. Recently, a scientific method combining single-cell analysis and microscopy has allowed for the simultaneous mapping of both the specialized diversity and spatial location of individual cells within a tissue or a tumor [134].

As mRNA populations may not always be fully reflected by the protein population, immunohistochemistry could better represent the cell situation at a given time and reflect protein cell diversity. For example, pyruvate carboxylase overexpression in PTC thyrocytes identifies the O_2_ reverse Warburg effect from the hypoxic Warburg metabolism of CAFs [56]. Immunohistochemistry, leading to cells’ individual oncogenic mutation determination through artificial intelligence methodology, could be one bridge between the two worlds: tissue vs. cells [133]. In 2021, Chervoneva et al. proposed an immunohistochemistry image-derived methodology on the spatial distribution of cellular signal intensity of protein expression within the breast cancer cell population, allowing to quantify both mean expression level and tumor heterogeneity [135]. Though all these methods allow to take into account tumor heterogeneity in regard to its spatial repartition, they remain an observation at a specific time point in tumor evolution.

### 4.2. Diagnosis and Cancer Heterogeneity

Early detection of cancer leads to increased opportunities for treatment options. Though many diagnostic techniques employed by pathologists and oncologists today have not changed over several decades, the advent of molecular technologies in diagnostics has allowed for the interrogation of a vast type of biological and clinical materials. If whole tissue from surgery can give a precise localized vision of the pathology or molecular biology (e.g., immunostaining, next generation sequencing, and RNA sequencing) of the tumor at one point in time, this does not apply to biopsies which are localized but offer a repetitive advantage to obtain information at different time points. However, false evaluation of cancer heterogeneity based on biopsy analysis, due to inappropriate pre-analytical conditions (tissue processing) have been recently described. Examples of such pre-analytical conditions include: (1) warm and cold ischemia affecting gene expression by inducing hypoxia, ischemia, and metabolic stress; (2) sample fixation time altering protein as well as nucleic acid integrity and detectability; and (3) tissue storage having possible implications for tumor molecular heterogeneity when comparison is required between samples related to different temporal evolutions of the pathological process. Considering a new type of tumor tissue sampling with standardized pre-analytical and analytical procedures will be paramount to evaluate heterogeneity [136].

Liquid biopsies which contain circulating tumor cells (CTCs) and other biomarkers such as tumor nucleic acids and exosomes have gained much attention over the past decade. They can be easily performed on various biofluids including blood, saliva, and cerebrospinal fluid and provide molecular information about cancer heterogeneities and emerging resistance mechanisms. For example, as both primary and metastatic tumors shed CTCs, a number of studies have investigated their molecular profiles to obtain insights in spatial heterogeneity. Moreover, monitoring the dynamic cellular changes in CTCs over time could allow for the assessment of temporal heterogeneity [137].

Along the same lines, as RNA-seq and similar methods that record gene expression within and among cells of homogenized biopsies lose positional information, Ståhl et al. developed spatial transcriptomics to overcome such issues. This method allows both visualization and quantitative analysis of the transcriptome with spatial resolution in individual tissue sections [138,139]. However, it is important to note that no method currently provides the depth of the transcriptome as does single cell RNA-seq, which underscores the need to combine single-cell and spatial data methodologies [140].

The last decade has witnessed the development of methods that try and capture the extensive intratumoral heterogeneity. On the one hand, a novel in vitro model of culture (organoids) to understand cancer has emerged in recent years. Organoids are complex, three-dimensional ex vivo tissue cultures that are derived from tissue or liquid biopsies. They represent a self-organized near-physiological model to study tumor cell heterogeneity and metastasis, as well as the systems involved and therapy response [141,142,143]. In addition to organoids, patient-derived xenograft models ease functional analysis for drug efficacy in solid tumors. On the other hand, the evolution of non-invasive imaging methods has permitted the study of cancer metabolism (i.e., metabolomics studies) or structure. Firstly, we can cite fluorodeoxyglucose-positron emission tomography (FDG-PET) imaging which is used in routine to detect increased glucose uptake by advanced/aggressive tumors such as breast, colorectal, and thyroid cancer (DTC, MTC, and ATC) [144,145,146]. In addition to FDG, a wide range of radiolabeled carbohydrates, amino acids, and fatty acids detected by PET are studied to try and take advantage of the high metabolic rate of tumors, creating a bridge between cell biochemistry and in vivo diagnostics [147,148]. Secondly, optical coherence tomography (OCT) is currently used for both preoperative diagnosis and intraoperative detection of some cancers such as skin, lung, and breast malignancies [149].

Finally, advances in material science, chemistry, engineering, and artificial intelligence could enable automated analysis to assist pathologists in diagnosis, and even in predicting survival outcomes. Perhaps, as mentioned before, histopathology analysis by deep learning will provide unbiased automated indications for therapy. A neural network trained to accurately and automatically classify whole-slide images into the two main lung cancer subtypes or normal-tissue, was developed [133] and can potentially be applied to any cancer type.

However, despite these methods, cancer heterogeneity poses a challenge under constant evolution for diagnosis. Indeed, ITH implies that the diagnosis of a tumor can evolve with time, inducing changes in patient treatment and survival.

### 4.3. Therapeutic Approaches vs. Cancer Heterogeneity

The extreme heterogeneity in time and space of the cancers casts some doubt on the validity of diagnoses and treatments based on local biopsies targeting defined precise oncogenic mutations. Of course, general initial genetic oncogenic mutations, even in 30% of the cells of a papillary carcinoma, suffice for a diagnosis. However, for functional molecular biology, e.g., gene expression, results for samples containing 20 to 30% cancer cells may be misleading in terms of interpretation, and therefore for adequate therapeutic options. Indeed, several studies demonstrate the need to overcome issues related to tumor heterogeneity prior to developing precision oncology strategies by showing that multiple regions of the same tumor can present different morphological and genetic features [150,151]. Moreover, the relatively slow-cycling, low-frequency stem cell phenotype populations may be those most involved in recurrences [152]. Likewise, mutational load may also vary between metastasis and its related primary tumor, but also in different metastasis originating from the same primary tumor [153]. In addition, different mutations in the same gene may have divergent effects in different cells, and the same mutation could have divergent effects in different tumors and even in the same tumor at distinct moments of its evolution [154]. Indeed, blocking HER2 in breast cancer has shown significant improvement in clinical outcomes, but did not confer the same results in the treatment of gastric cancer, for example [155]. Likewise, the same treatment could have a different impact during the evolution of the tumor. For instance, in a tumor, the same antioxidants would be beneficial at an early stage, but detrimental at a later stage [88].

Tumor heterogeneity is often accompanied by qualitatively different drug responses [156]. Indeed, due to tumor heterogeneity and molecular mechanisms of resistance, precision medicine could easily target an irrelevant part of the tumor unless a primary mutation present in several parts of the tumor is targeted. As a result of tumor heterogeneity, most of the targets considered as druggable are not expressed homogeneously within the tumor. For instance, endocrine therapy, such as Fulvestrant or Tamoxifen, is an effective targeted therapy for ER-positive breast cancer. However, its activity depends on the fraction of ER positive cells within a tumor which can widely vary between entities [157]. Such considerations could raise some doubts about the fashion of personalized medicine attacking the right target for each cancer, when they are in fact diverse moving targets in the same tissue [158]. In addition, intratumor heterogeneity, associated with heterogeneous protein function, may foster tumor adaptation and therapeutic failure through “Darwinian evolution”. This point has been emphasized for thyroid [13], renal carcinoma [151], and mammary tumors [159]. Indeed, the progression-free survival of many cancers after specific drug treatment, e.g., of such treated small-cell lung cancers, are not impressive [160,161]. Several molecular mechanisms which confer therapeutic resistance at both cellular and TME levels have been described. (1) drug sequestration inside the cell (endosome) or in the extracellular area (exosome), which can entrap compounds by means of transporters or by specific binding, have been described. For example, HER2-containing exosomes released by tumor cells bind trastuzumab and reduce the amount of the therapeutic agent that can interact with cancer cells [162]. (2) Some cancer cells can have an increased expression of ABC transporters which generate an efflux of the drug towards the extracellular area. (3) Due to the cancer cells’ ability to escape apoptosis (by mutation or through TME cell assistance), drugs favoring this pathway become inefficient. Moreover, as previously mentioned, the TME also plays an important role in drug resistance [163]. As examples we can cite, (1) survival cytokines, growth factors, and exosomes sent by CAFs allowing cells to share material (e.g., miRNAs), thus spreading resistance to sensitive cancer cells [164]; (2) The extracellular matrix generates a real barrier using elements from the TME, preventing drugs from reaching the cancer cells; (3) Hypoxia generated in the TME can affect chemical drugs’ efficacy by preventing their transport across the membrane due to the pH gradient between the extracellular acidic pH and the almost-neutral intracellular pH; (4) TAMs favor an immunosuppressive environment, reducing the sensitivity of the tumor to cancer immunotherapy [163].

The general observation that, in the absence of a functional immune system, many tumors develop, points to immunotherapy as the natural therapeutic tool. For instance, the anti-PD1 and anti-PD1L immunology treatments are more and more considered as first-line and general treatments [165,166]. Harnessing innate immunity is another approach [167]. However, in this field, heterogeneity will also play a role; thus, there are many relevant approaches which have been recently reviewed by Vitale et al. [168]. Another important target for immunotherapy are neoantigens, owing to their strong immunogenicity and lack of expression in normal tissues. Indeed, therapeutic vaccines have been carried out in three human studies for advanced melanoma with strong and long-lasting anti-tumor effects reported [169,170]. However, though tumor-derived neoantigens may represent new therapeutic opportunities, the clonal heterogeneity of neoantigenic architecture raises new challenges [171].

Finally, new approaches as synthetic lethality, i.e., the use of known, no longer patented drugs acting on different complementary targets, in combinations at low dosage, offer a new, inexpensive, and very promising general perspective of therapy [172,173].

## 5. Conclusions

In recent decades, our ability to detect cancer heterogeneity and our understanding of its importance have evolved. Indeed, technological innovations illustrate cancer heterogeneity, leading to the concept of cancer as an independent cooperative heterogeneous cell population with its own independently evolving biology; almost an organism. A better understanding of this “organism” and the communication between cells implies gathering an important quantity of information from the combination of “omics” technics (e.g., genomic, metabolomics), histologic features, and drug efficacy testing (e.g., organoids), among others. Combining previous and new technologies allowing to assess spatial (e.g., spatial transcriptomics, machine learning) and temporal heterogeneity (e.g., liquid biopsies) will contribute to further refine knowledge on cancer heterogeneity, and could prove useful to overcome the limitations of precision medicine, as well as for new diagnostic approaches. Not only should this massive data be combined and require discussion and translation of the molecular profiles into clinical benefit for the patients, but dynamic heterogeneity of cancer should lead to a reconsideration and constant reevaluation of present habits and fashions in concepts, diagnosis and therapy.
